# Kupffer Phase Radiomics Signature in Sonazoid-Enhanced Ultrasound is an Independent and Effective Predictor of the Pathologic Grade of Hepatocellular Carcinoma

**DOI:** 10.1155/2022/6123242

**Published:** 2022-06-27

**Authors:** Chen Li, Jingyong Xu, Yuan Liu, Mingxiao Wu, Weide Dai, Jinghai Song, Hanzhang Wang

**Affiliations:** ^1^Department of Ultrasound, Beijing Hospital, National Centre of Gerontology, Institute of Geriatric Medicine, Chinese Academy of Medical Sciences, Beijing, China; ^2^Department of General Surgery and Hepato-Bilio-Pancreatic Surgery, Beijing Hospital, National Centre of Gerontology, Institute of Geriatric Medicine, Chinese Academy of Medical Sciences, Beijing, China; ^3^GE Healthcare, Shanghai, China

## Abstract

We conduct this study to investigate the value of Kupffer phase radiomics signature of Sonazoid-enhanced ultrasound images (SEUS) for the preoperative prediction of hepatocellular carcinoma (HCC) grade. From November 2019 to October 2021, 68 pathologically confirmed HCC nodules from 54 patients were included. Quantitative radiomic features were extracted from grayscale images and arterial and Kupffer phases of SEUS of HCC lesions. Univariate logistic regression and the maximum relevance minimum redundancy (MRMR) method were applied to select radiomic features best corresponding to pathological results. Prediction radiomic signature was calculated using each of the image types. A predictive model was validated using internal leave-one-out cross validation (LOOCV). For discrimination between poorly differentiated HCC (p-HCC) and well-differentiated HCC/moderately differentiated HCC (w/m-HCC), the Kupffer phase radiomic score (KPRS) achieved an excellent area under the curve (AUC = 0.937), significantly higher than the other two radiomic signatures. KPRS was the best radiomic score based on the highest AUC (AUC = 0.878), which is prior to gray and arterial RS for differentiation between w-HCC and m/p-HCC. Univariate and multivariate analysis incorporating all radiomic signatures and serological variables showed that KPRS was the only independent predictor in both predictions of HCC lesions (p-HCC vs. w/m-HCC, log OR 15.869, *P* < 0.001, m/p-HCC vs. w-HCC, log OR 12.520, *P* < 0.05). We conclude that radiomics signature based on the Kupffer phase imaging may be useful for identifying the histological grade of HCC. The Kupffer phase radiomic signature may be an independent and effective predictor in discriminating w-HCC and p-HCC.

## 1. Introduction

Liver cancer is the sixth most common malignancy and the fourth most common cause of cancer-related death worldwide [1]. Hepatocellular carcinoma (HCC) represents primary liver cancer and is the second leading cancer-related mortality in China [2]. Clinically, the development of HCC is prone to metastasis and recurrence, which limits the prognosis as well as the quality of life [3]. Pathological grading is associated with intrahepatic recurrence [3]. High-grade HCC tumors have a higher risk of intrahepatic recurrence than low-grade tumors [4]. Accurate prediction of the HCC grade of differentiation might formulate the treatment strategy and predict the therapeutic outcome, prognosis, and risk of tumor recurrence [5, 6].

Medical radiological imaging is integral to the routine clinical method of patients with HCC. Radiomics is a technology that extract the characteristics of radiological image quantitatively [7]. Conventional imaging evaluation provides few metrics without tumor heterogeneity information through the manual assessment of lesions by radiologists [8]. With the development of medical imaging data, radiomics are used to deeply excavate the biological characteristics of tumor imaging, quantitatively analyze tumor heterogeneity, and integrally evaluate tumor phenotype, which may be beyond conventional techniques. In fact, recent studies have developed that pathological grading is related to the radiomics algorithm acquired from magnetic resonance imaging (MRI) or computed tomography (CT), such as prediction of a pathological grade of gliomas and renal carcinoma [9, 10].

Compared with CT and MRI, ultrasound (US) is simple, radiation-free, inexpensive, and commonly used to monitor the liver lesions [11]. Contrast-enhanced ultrasound score (CEUS) can observe the real-time vascular phase with microcirculation perfusion information. The microbubbles of a contrast agent called Sonazoid can be phagocytosed by Kupffer cells, which rarely exist in tumors, and then Sonazoid-enhanced US (SEUS) provides a special phase called the Kupffer phase or the post-vascular phase [12, 13]. Previous studies have reported that the degree of the contrast defect in the Kupffer phase are related to histological grading of HCC, and certain quantifiable patterns of CEUS were associated with treatment outcomes [14–16].

To better interpret SEUS, we have, therefore, developed radiomics for evaluating the histological grading of HCC based on US, arterial phase, and Kupffer-phase by SEUS. Our study aims to evaluate the feasibility of US and SEUS radiomics models in terms of differentiation histologically grades of HCC to determine an initial prognosis of HCC.

## 2. Materials and Methods

### 2.1. Patients

**Figure 1:**
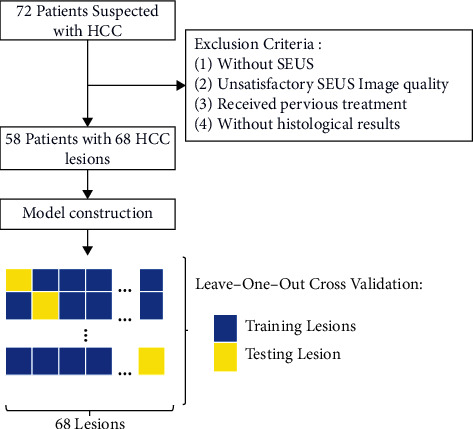
Flowchart of the study.

The institutional review board of our institution approved this retrospective study and waived the requirement to obtain written informed consent.  Figure 1 shows the enrolment of patients.

Between November 2019 and October 2021, 71 consecutive patients with HCC were retrospectively recruited. Inclusion criteria were as follows: (1) grayscale ultrasound image and SEUS were performed preoperatively in each patient; (2) no prior surgical or medical treatment was administered for the suspected HCC lesions; (3) the diagnoses of HCC were obtained by US/CT-guided percutaneous biopsy and surgical resection. Exclusion criteria were as follows: (1) patients without SEUS; (2) patients with previous treatment (i.e., radiofrequency ablation or trans-arterial chemoembolization) before ultrasound imaging; (3) patients without available histological report; (4) unclear or unsatisfied grayscale or SEUS images of focal liver lesions. A total of 54 patients with 68 histologically confirmed HCC nodules were enrolled in this study. The clinical characteristics of patients contain age, gender, tumor maximum diameter, alpha-fetoprotein (AFP) values, total bilirubin (TBil), direct bilirubin (DBil), carbohydrate antigen 12-5 (CA12-5), carbohydrate antigen 19-9 (CA19-9), carbohydrate antigen 15-3 (CA15-3), aspartate transaminase (AST), and alanine transaminase (ALT).

### 2.2. Assessment of Pathological Grade

Among 54 patients with 68 HCC nodules, the diagnosis was confirmed by pathological examination of specimens obtained by US/CT-guided percutaneous biopsy (*n* = 28) and surgical resection (*n* = 41). Histological grade of HCC tumors was obtained by the pathologist. According to the International Working Party Classification, the degree of differentiation was determined [7]. Histological grade included well-differentiated HCC (w-HCC, *n* = 12, 17.4%), moderately differentiated HCC (m-HCC, *n* = 41, 59.4%), and poorly differentiated HCC (p-HCC, *n* = 15, 21.7%).

### 2.3. Contrast-Enhanced Ultrasound Imaging

All patients underwent conventional ultrasound in B-mode and SEUS by two sonographers with more than 5 years of experience in standard liver CEUS. All SEUS was performed by two sonographers using Aplio 500 (Canon, Honshu, Japan) with a convex probe (6C1, 1–6 MHz) and a linear probe (11L4, 4–11 MHz) and Aplio i800 (Canon, Honshu, Japan) with a convex probe (PVI-475BX, 1–8 MHz) and a linear probe (11L4, 4–11 MHz). The mechanical index (MI) for the acoustic output was set to 0.19–0.22 and the dynamic range was 65–70 dB according to the size of the lesion. Patients received a bolus intravenous injection of Sonazoid (perfluorobutane, GE Healthcare, Oslo, Norway) through a peripheral venous line, followed by 5 mL of normal saline flush. Immediately after the administration of Sonazoid, the hepatic arterials, portal veins, hepatic veins, and the normal liver parenchyma were uniformly enhanced during an early vascular phase image lasting 3 minutes. Approximately 10 minutes after injection, the liver was scanned again to observe the post-vascular phase image (Kupffer phase). The arterial phase and the Kupffer phase were obtained by scanning 15 to 30 seconds and 15 minutes, respectively.

### 2.4. Image Segmentation

Image segmentation was performed on grayscale, arterial phase, and Kupffer phase retrieved from DICOM (Digital Imaging and Communications in Medicine) format files. The images were loaded into the ITK-SNAP software (open-source software, https://www.itksnap.org) for manual segmentation, and a three-dimensional volume of interest (VOI) that covered the whole tumor was delineated in the images, respectively, segmented by a sonographer with over five years of experience in abdominal CEUS imaging. The procedure is shown in Figure 1 and details are introduced as follows.

### 2.5. Radiomics Analysis

After integrating VOI that covered the whole tumor images, a three-dimensional radiomics feature was extracted from grayscale, arterial phase, and Kupffer phase images using the NUK software (novo ultrasound kit, GE Healthcare Shanghai, China). Specifically, shape-based (*n* = 9), first-order (*n* = 18), gray-level cooccurrence matrix (GLCM, *n* = 24), gray-level dependency matrix (GLDM, *n* = 14), gray-level run-length matrix (GLRLM, *n* = 16), gray level size zone matrix (GLSZM, *n* = 16), and neighbouring gray tone difference matrix (NGTDM, *n* = 5) features, according to the imaging biomarker standardization initiative from both original images and filter derived images were extracted. SMOTE up- and down-sampling was applied to create a balanced training dataset, which had been used in other radiomic studies.

Z-score normalization was applied to radiomic features. Each radiomic feature's association with the outcome (pathologically confirmed HCC differentiation status) were initially assessed using univariate logistic analysis. Radiomic features significant in univariate analysis were further selected using maximum relevance minimum redundancy (MRMR) to obtain 15 features most contributing to the outcome with least correlation. The final prediction of the outcome was obtained by a random forest classifier (RFC) trained on selected radiomic features, in the form of a radiomic score per lesion, which was calculated by linear combination of radiomic features with associated weights.

The univariate-multivariate logistic model with an adjusted odds ratio (OR) was constructed using the radiomic score calculated from grayscale radiomic score (grayRS), arterial phase radiomic score (APRS), and Kupffer phase radiomic score (KPRS) images. Different HCC differentiations (low, medium, and high) were analyzed using “one versus rest” strategy (OvR).

**Figure 2:**
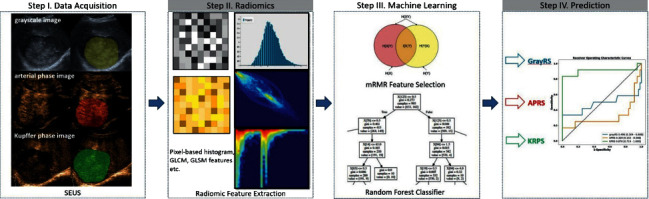
Radiomics analysis. SEUS, Sonazoid-enhanced ultrasound; grayRS, grayscale radiomic score (grayRS); APRS, arterial phase radiomic score; KPRS, Kupffer phase radiomic score.

Radiomic scores and the model's discrimination ability of pathological HCC differentiation were characterized using receiver operation characteristic (ROC) analysis; the area under the curve (AUC) was used to quantify model performance. Predictive performances including accuracy, sensitivity, and specificity of the predictors were calculated at the optimal decision boundary on the ROC curve determined by maximizing the Youden's index. Leave-one-out cross validation (LOOCV) was applied for model validation (Figure 2).

### 2.6. Statistical Analysis

Descriptive statistics were presented in mean with standard deviation or median with interquartile rage depending on variables' normality. The Shapiro–Wilk test was used to asses normality. 95% Confidence intervals for model evaluation were calculated using the bootstrap method with 1000 random resamples. The DeLong test was used to compare AUC differences. The McNemar Chi-squared test was used to compare predictive performances. The Hosmer-Lemeshow test was used to assess significance of model's deviation from perfect fit. Variables significant in univariate analysis were passed to multivariate analysis. A two-sided *P* value less than 0.05 was considered statistically significant.

## 3. Results

### 3.1. Patient Characteristics

In total, 54 patients were included containing 48 males and 6 females, with a mean age of 61.5 years (range from 41 to 88 years). Sixty-eight lesions were detected and studied among 54 HCC patients. Most of the patients had a history of chronic liver disease and cirrhosis, including hepatitis C virus (HCV) infection in 1 patient (1.9%), hepatitis B virus (HBV) infection in 43 patients (79.6%), HCV and HBV infection in 3 patients (5.6%), nonalcoholic steatohepatitis (NASH) in 7 patients (13%), and cirrhosis in 40 patients (74.1%). The median total bilirubin and direct bilirubin were 14.70 (10.00–21.20) and 5.00 (3.70–7.80) *μ*mol/L, respectively. ALT was 24.00 (17.75–49.75) and AST was 30.00 (21.00–55.50) U/L. Some of the tumor markers were recorded, such as AFP 5.90 (2.85–122.45) *μ*g/L, CA19-9 8.75 (3.92–17.15) U/ml, CA125 14.35 (9.47–30.93) U/ml, and CA153, 8.70 (6.65–11.93) U/ml.

### 3.2. Differentiation of w/m-HCCs from p-HCCs

**Figure 3:**
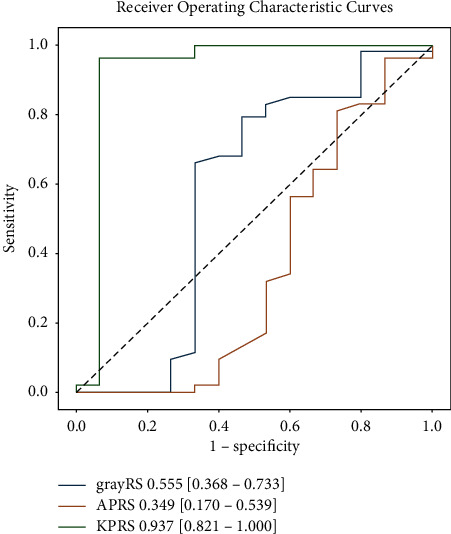
ROC curves of radiomic features: the differentiation between p-HCC and w/m-HCC.

At the task of differentiating w/m-HCC from p-HCC lesions, radiomic scores calculated from grayRS and APRS displayed poor if not no discrimination abilities based on ROC analysis. KPRS showed an excellent AUC of 0.937 (95% confidence interval (CI): 0.821, 1.000), significantly higher than the other two radiomic scores (All DeLong test *P* < 0.05). KPRS achieved an accuracy, sensitivity, and specificity of 97.1% (95% CI: 92.6%, 100%), 93.3% (80.0%, 100%), and 98.1% (94.6%, 100%), respectively (Table 1, Figure 3).

Univariate analysis showed that no serological variables were significantly related to p-HCC and w/m-HCC (Table 2). APRS (log OR −6.533, 95% CI (−11.339, −1.728), *P* < 0.05) and KPRS (log OR 14.940, 95% CI (7.313, 22.567), *P* < 0.001) showed significance in univariate analysis, but only KPRS demonstrated to be an independent predictor in multivariate analysis (log OR 15.869, 95% CI (7.218, 24.520), *P* < 0.001).

### 3.3. Differentiation of w-HCC from m/p-HCC

**Figure 4:**
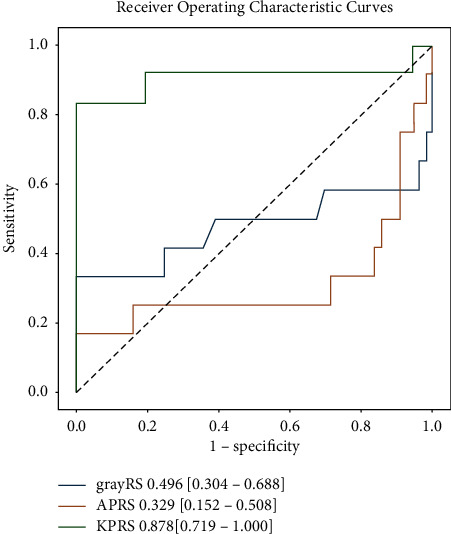
ROC curves of radiomic features: the differentiation between w-HCC and m/p-HCC.

KPRS was the best radiomic score based on the highest AUC: 0.878 (95% CI: 0.719, 1.000). KPRS also showed excellent predictive performance (Table 3, Figure 4) with a sensitivity of 83.3% and specificity of 100%. As a comparison, grayRS and APRS displayed AUC lower than 0.5, which was considered poor performance.

Univariate analysis showed that CA19-9 (log OR 0.016, 96% CI (0.004, 0.029), *P* < 0.05) and KPRS (log OR 14.454, 95% CI 7.154, 21.753), *P* < 0.001) were significant predictors for differentiation w-HCC from m/p-HCC (Table 4). KPRS (log OR 12.520, 95% CI (5.333, 19.707), *P* < 0.05) was the only independent predictor in multivariate analysis. KPRS was sufficient at discriminating w-HCC and m/p-HCC.

## 4. Discussion

Radiomics is a noninvasive technology based on the quantitative extraction of signature from radiological imaging modalities [7]. In fact, investigators have shown that radiomics may be useful for predicting progression-free and overall survival for malignant diseases [17, 18]. Recently, radiomics analysis based on ultrasound imaging technology has achieved some good results in the early diagnosis, prognosis, and prediction of diseases.

CEUS is widely used to observe microcirculation blood perfusion of liver cancer [19]. We used the radiomics method to evaluate the overall information related to the difference of grade that maybe contained in tumors by extracting multiphase CEUS imaging features. Therefore, the aim of our study was to develop and validate CEUS radiomics models based on US, arterial phase and Kupffer-phase for predicting the histological grading of HCC. Our study showed that for discrimination between p-HCC and w/m-HCC, KPRS showed an excellent AUC of 0.937, significantly higher than grayRS and APRS. Meanwhile, KPRS was the best radiomic score based on the highest AUC (AUC = 0.878), which is prior to grayRS and APRS for the differentiation between w-HCC and m/p-HCC. Wu et al. investigated MRI-based radiomics signatures for the HCC grade, and the AUC of model using radiomics signatures was 0.742 [20]. Our study showed that the prediction model using radiomics signatures based on KPRS (AUC = 0.937, 0.878) is prior to MRI, which means KPRS had advantages in predicting the HCC grade. The easy-to-use graphic tool might provide important characteristics to stimulate clinical prediction. Therefore, our study had potential application of SEUS in the diagnosis of focal liver lesions than conventional contrast medium and CT [21]. Moreover, with the number of focal liver lesions increased in HCC and other kind of tumor prior to different subtypes of hepatocellular adenoma [22].

Our study demonstrated that only KPRS demonstrated to be an independent predictor in univariate analysis and multivariate analysis in predicting the HCC grade. On the other hand, KPRS showed a better discrimination performance compared with the combination of clinical risk factors and KPRS, while, the results of were inconsistent with the previous studies. Wu et al. showed that the combination of MRI radiomics signatures with clinical factors could be useful for discriminating between high-grade and low-grade HCC, and both the AFP level and radiomics signatures were independent predictors [20]. Mohamed et al. demonstrated that serological markers, such as serum vitronectin and AFP, speculated a potential role in diagnosis and prognosis of HCC [23]. This is, probably, because of the different classification of pathological grade and the number of cases, HCC tumors were divided into low-grade and high-grade cases instead of using the International Working Party Classification or the Edmondson grade.

Limitations of this study should be acknowledged. First, the number of HCC cases was relatively limited, and HCC tumors were divided into well, moderate, and poorly differentiate-cases, while no ideal results were obtained discriminating between m-HCC and rest. Second, our study was performed in a single center, further multicenter cohorts might be necessary to evaluate the reliability, and to verify the generalizability of our findings. Third, the potential use of SEUS combined with gadoxetic acid-enhanced magnetic resonance may provide more characteristics to increase prediction [24]. In the future, multimodality ultrasound imaging-including color Doppler-flow imaging, ultrasound elastography, and vascular phase of CEUS imaging-will be combined to improve the performance of HCC classification.

## 5. Conclusions

In conclusion, radiomics signatures based on the Kupffer phase imaging may be useful for identifying the histological grade of HCC. Additionally, the Kupffer phase radiomic signature may be an independent and effective predictor in discriminating w-HCC and p-HCC.

## Figures and Tables

**Table 1:** Prediction performance on p-HCC versus w/m-HCC.

Radiomic score	AUC	Accuracy	Sensitivity	Specificity
grayRS	0.555 (0.368, 0.733)	66.2% (55.9%, 75.0%)	46.7% (23.8%, 66.7%)	71.7% (60.4%, 81.5%)
APRS	0.349 (0.170, 0.539)	79.4% (72.1%, 86.8%)	13.3% (0.00%, 30.0%)	98.1% (94.4%, 100.0%)
KPRS	0.937 (0.821, 1.000)	97.1% (92.6%, 100.0%)	93.3% (80.0%, 100.0%)	98.1% (94.6%, 100.0%)

grayRS : grayscale radiomic score; APRS : arterial phase radiomic score; KPRS : Kupffer phase radiomic score.

**Table 2:** Univariate analysis and multivariate analysis for p-HCC versus w/m-HCC.

Variables	Univariate				Multivariate			
	log (OR)	(0.025	0.975)	*P*	log (OR)	(0.025	0.975)	*P*
AFP	0.000	0.000	0.000	1.000				
TBil	0.001	−0.007	0.009	0.788				
DBil	0.000	−0.007	0.008	0.951				
AST	0.004	0.000	0.010	0.263				
ALT	0.005	−0.003	0.012	0.195				
CA125	−0.001	−0.005	0.002	0.451				
CA19-9	0.003	−0.007	0.013	0.606				
CA153	−0.011	−0.061	0.040	0.671				
grayRS	0.145	−4.763	5.054	0.954				
APRS	−6.533	−11.339	−1.728	0.023	−9.132	−18.686	0.422	0.061
KPRS	14.940	7.313	22.567	<0.001	15.869	7.218	24.520	<0.001
const.					−1.363	−3.693	0.968	0.252

AFP, alpha-fetoprotein; TBil, total bilirubin; DBil, direct bilirubin; AST, aspartate transaminase; ALT, alanine transaminase; CA, carbohydrate antigen; grayRS, grayscale radiomic score; APRS, arterial phase radiomic score; KPRS, Kupffer phase radiomic score; const., constant.

**Table 3:** Prediction performance on w-HCC versus m/p-HCC.

Radiomic score	AUC	Accuracy	Sensitivity	Specificity
GrayRS	0.496 (0.304, 0.688)	85.3% (77.9%, 91.2%)	25.0% (7.1%, 45.5%)	98.2% (94.7%, 100.0%)
APRS	0.329 (0.152, 0.508)	82.4% (75.0%, 89.7%)	16.7% (0.00%, 35.7%)	96.4% (92.5%, 100.0%)
KPRS	0.878 (0.719, 1.000)	97.1% (92.6%, 100.0%)	83.3% (62.5%, 100.0%)	100.0% (100.0%, 100.0%)

grayRS: grayscale radiomic score; APRS: arterial phase radiomic score; KPRS: Kupffer phase radiomic score.

**Table 4:** Univariate analysis and multivariate analysis m/p-HCC versus w-HCC.

Variables	Univariate				Multivariate			
	log (OR)	(0.025	0.975)	*P*	log (OR)	(0.025	0.975)	*P*
AFP	0.000	0.000	0.000	1				
TBil	0.009	−0.005	0.023	0.224				
DBil	0.009	−0.006	0.023	0.226				
AST	−0.001	0.007	0.004	0.641				
ALT	−0.003	−0.010	0.003	0.327				
CA125	0.000	−0.006	0.005	0.86				
CA199	0.016	0.004	0.029	0.007	0.011	−0.009	0.031	0.269
CA153	0.009	−0.045	0.063	0.755				
grayRS	−3.165	−8.381	2.052	0.234				
APRS	−1.813	−4.792	1.166	0.233				
KPRS	14.454	7.154	21.753	<0.001	12.520	5.333	19.707	0.017
const.					−11.093	−17.036	−5.151	<0.001

AFP, alpha-fetoprotein; TBil, total bilirubin; DBil, direct bilirubin; AST, aspartate transaminase; ALT, alanine transaminase; CA, carbohydrate antigen; grayRS, gray scale radiomic score; APRS, arterial phase radiomic score; KPRS, Kupffer phase radiomic score; const., constant.

## Data Availability

The data that support the findings of this study are available from the corresponding author upon reasonable request.
